# A baculovirus dual expression system-based vaccine confers complete protection against lethal challenge with H9N2 avian influenza virus in mice

**DOI:** 10.1186/1743-422X-8-273

**Published:** 2011-06-04

**Authors:** Wenyao Lin, Huiying Fan, Xiaoliang Cheng, Yu Ye, Xiaowei Chen, Tao Ren, Wenbao Qi, Ming Liao

**Affiliations:** 1 Key Laboratory of Animal Disease Control and Prevention of the Ministry of Agriculture, Guangzhou 510642, China; 2 College of Veterinary Medicine, South China Agricultural University, Guangzhou 510642, China

**Keywords:** Avian influenza viruses, H9N2, vaccine, HA protein, Baculovirus dual expression system, immune response

## Abstract

**Background:**

Avian influenza viruses of H9N2 subtype have become highly prevalent in avian species. Although these viruses generally cause only mild to moderate disease, they can infect a wide variety of species, including chickens, quail, turkeys, ducks, geese, pheasant, partridge, and pigeon, even transmitted to mammalian species, including humans, accelerating the efforts to devise protective strategies against them.

**Results:**

The results showed that stronger immune responses were induced in a mouse model immunized with BV-Dual-HA than in those vaccinated with a DNA vaccine encoding the same antigen. Moreover, complete protection against lethal challenge with H9N2 virus was observed in mice.

**Conclusion:**

BV-Dual-HA could be utilized as a vaccine candidate against H9N2 virus infection.

## Background

Influenza A viruses of the H9N2 subtype have become highly prevalent in poultry in many countries, and although these viruses generally cause only mild to moderate disease, they can infect a wide variety of species, including chickens, quail, turkeys, ducks, geese, pheasant, partridge, and pigeon [1-4]. More importantly, occasional transmission of H9N2 viruses from land-based poultry to humans and pigs have been reported [5-7]. Some investigations suggest that a significant proportion of H9N2 field isolates have acquired human virus-like receptor specificity; a few that could recognize α 2,6-linked sialic acid (SAα2-6) have been transmitted directly to humans [7-10]. In addition to possessing human virus-like receptor specificity, avian H9N2 viruses induce a typical human flu-like illness, which can easily go unreported, and therefore have the opportunity to circulate, reassort, and improve transmissibility [7,11-14]. Hence, global concern is focused on the prevention and treatment of H9N2 avian influenza virus infections.

Prevention of avian influenza is mainly through vaccination. Currently, most avian influenza vaccines used in clinics are the inactivated type, which are propagated in embryonated chicken eggs. However, the use of inactivated avian influenza vaccines can induce little or no cellular immune response; thus, it cannot provide wide and persistent protection against influenza, and it will interfere with serological monitoring. In addition, egg-based influenza vaccine production is dependent on the availability of embryonated eggs, which is at risk in the event of outbreaks of avian diseases. In view of these potential drawbacks, we sought to develop a new type of H9N2 vaccine using the Baculovirus Dual Expression System constructed in this study.

The baculovirus *Autographa californica *multiple nucleopolyhedrovirus (AcMNPV) has traditionally been an excellent tool to overexpress recombinant proteins in insect cells. Since the discovery that baculovirus is capable of entering mammalian cells and mediating transgene expression under the promoter active in mammalian cells [15], baculoviral vectors have been exploited as versatile vaccine vehicles to produce vaccine candidates against different pathogens.

Recently, AcMNPV has been further engineered for application as a new eukaryotic display system to express foreign proteins on the surface of the viral envelope [16-20] and formed a hedgehog-shaped "fake virus". This display system relies on the main envelope protein of AcMNPV gp64 protein, which causes the surface display of foreign proteins on the baculovirus surface. This method has been extended to develop pseudotype baculoviruses as a potential vaccine delivery platform. Several research groups have demonstrated that direct vaccination with this kind of pseudotype baculoviruses can induce high titers of antigen-specific antibodies [17,21].

Subsequently, it was determined that some natural viral envelope proteins such as influenza hemagglutinin (HA) can be displayed on the baculovirus surface, even without fusion with gp64. Tang et al. [20] and Yang et al. [19] reported that signal peptide (SP) and cytoplasmic tail (CT) domains of gp64 can enhance the display of HA on the viral surface, while the transmembrane (TM) domain of gp64 impairs HA display. Therefore, a chimeric HA with SP and CT derived from gp64 was chosen for our study.

Combining the characteristics of baculovirus as a gene delivery vehicle and surface display system, we constructed a "Baculovirus Dual Expression System," (BV-Dual-HA), which is capable of displaying H9N2-HA protein on the surface of the viral envelope and expressing it upon transduction in mammalian cells. The main objectives of this study were: i) to effectively display functional HA on the baculoviral envelope in the hope that HA would retain superior immunogenicity upon in vivo immunization, and ii) to efficiently express HA in transduced mammalian cells. The results showed that stronger immune responses were induced in a mouse model immunized with BV-Dual-HA than in those vaccinated with a DNA vaccine encoding the same antigen. Moreover, complete protection against lethal challenge with H9N2 virus was observed in mice, indicating the potential of BV-Dual-HA as a vaccine candidate against H9N2 virus infection.

## Materials and methods

### Animals

Six-week-old BALB/c female mice were purchased from Southern Medicine University, Guangzhou, China, and were housed, fed in microisolator units according to the Veterinary guidelines of South China Agricultural University and all animal experiments were approved by the South China Agricultural University Institutional Animal Care and Use Committee.

### Cell lines and virus

A porcine kidney PK-15 cell line free of PCV1 contamination (ATCC CCL 33) was maintained in Dulbecco's modified Eagle's medium (Invitrogen, USA) and supplemented with 10% (v/v) heat-inactivated fetal bovine serum (FBS; Invitrogen, USA), 100 μg/ml of streptomycin, and 100 IU/mL of penicillin. The *Spodoptera frugiperda *cells (sf9) used to propagate wild-type and recombinant baculoviruses were cultured in Grace's insect media (GIBCO; Invitrogen, USA) and supplemented with 10% heat-inactivated FBS at 27°C. MDCK cells were used to propagate avian influenza virus and were cultured in minimal essential medium (MEM) containing 10% fetal calf serum at 37°C.

The low-pathogenicity influenza A/Chicken/Guangzhou/V/2008(H9N2) virus was isolated in Guangdong Province, China. It is a mouse-adapted H9N2 strain that can cause infection and death in mice, and was used to challenge immunized mice in this study.

### Construction of recombinant baculovirus

To construct a baculovirus transfer vector, psurf-HA, a gene lacking the N-terminal SP and C-terminal CTD but encoding the ectodomain of HA, was amplified from pc-HA plasmid (a DNA vaccine was constructed with the pcDNA3.1(+) vector expressing H9N2 HA protein in our lab; Invitrogen) and inserted between the sequences encoding the gp64 signal peptide and gp64 cytoplasmic domains of a pBACsurf-1 vector (whose gp64 ectodomain and transmembrane domain were deleted earlier; Novagen). To construct pCMV-surf-HA, a 2.3-kb fragment of a chimeric HA gene was excised from psurf-HA by digestion with *Nhe*I and *Hind*III, and inserted into the *Nhe*I/*Hind*III sites of the pcDNA3.1(+) vector (Invitrogen). The cassette consisted of the CMV-IE promoter; chimeric HA gene was amplified by PCR and subcloned into a pFastBac™ plasmid with *Sal*I/*Hind*III treatment to generate BV-Dual-HA. The resultant BV-Dual-HA plasmid thus contained separate HA genes driven by CMV-IE and Pph promoters.

Recombinant baculovirus BV-Dual-HA was produced using the Bac-to-Bac^® ^system and was propagated in sf9 insect cells according to standard methods. Virus particles were purified by 2 rounds of sucrose gradient ultracentrifugation following standard protocols [22], and infectious titers were determined with the BD BacPAK baculovirus rapid titer kit (Clontech Laboratories, USA).

### Immunoelectron microscopy

Purified virus particles were absorbed onto carbon-coated copper grids and incubated with a monoclonal antibody (mAb) against HA of H9N2 (prepared in our lab) for 1 h. The grids were incubated with goat anti-mouse IgG labeled with 5-nm gold particles (Sigma) for 30 min. After 3 additional PBS washes, the grids were stained with 2% phosphotungstic acid (Sigma, St. Louis, MO, USA) and examined under transmission electron microscopy (H-7500; Hitachi, Tokyo, Japan).

### Indirect fluorescence assay

PK-15 cells were seeded at a concentration of 2.5 × 10^5 ^cells/well into 6-well tissue culture plates (Corning Costar Co., Cambridge, MA, USA) and transduced with purified baculovirus particles at an MOI of 10. After 48 h incubation, the cells were fixed with absolute methanol for 5 min at -20°C, rinsed with PBS, and blocked with 2% bovine serum albumin for 30 min at 37°C. The cells were then incubated with the primary anti-body (anti-HA mAb) for 1 h at 37°C, followed by 3 PBS washes. The cells were then incubated with the secondary antibody (FITC-conjugated rabbit anti-mouse IgG; Sigma) for 1 h at 37°C, followed by 3 PBS washes. Fluorescence images were examined under an inverted fluorescence microscope (Olympus IX70).

### Animal experiments

Six-week-old BALB/c female mice were randomly divided into four groups, each containing 12 mice. Three groups were vaccinated intramuscularly (i.m.) with 10^9 ^PFU of BV-Dual-HA, 10^9 ^PFU of wild-type AcMNPV (AcMNPV-WT), and 100 μg of pc-HA, respectively. On days 0 and 21. The final group was used as the control and injected with 100 μL PBS. Serum samples were collected on days 20 and 42 for serological tests. On day 42, the mice were challenged intranasally (i.n.) with 50 MLD_50 _(50% mouse lethal dose) of influenza A/Chicken/Guangzhou/V/2008(H9N2) and observed for clinical signs over a 14-day period. Mice were weighed daily and examined for disease. Mice that lost more than 20% body weight were humanely euthanized. Six days after challenge, 3 mice from each group were sacrificed and the lungs, brains, livers, kidneys, and spleens were harvested to examine virus replication in SPF embryonated eggs. The viral titer, expressed as EID_50 _(50% egg infection dose), was calculated by the Reed-Muench method.

### Serological testing

The hemagglutination inhibition (HI) assay and virus neutralization (VN) assay were performed as described previously [23].

### Statistical analysis

An analysis of variance and Student's *t*-test were used to evaluate potential differences among the different groups with regard to the humoral immune responses, viral burdens, and body weights. Differences between groups were considered significant at P < 0.05.

## Results

### Construction of baculovirus dual expression system

**Figure 1:**
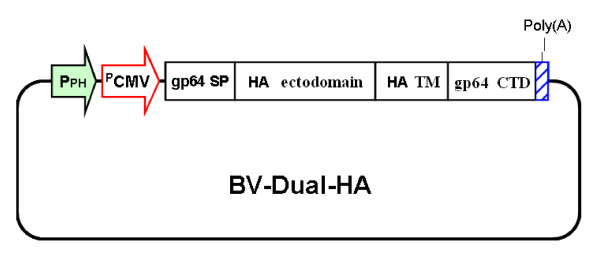
**Schematic representation of BV-Dual-HA structure**. The gene cassette consists of the gp64 signal peptide (SP), the HA ectodomain gene, the HA transmembrane domain (TM), gp64 cytoplasmic domain (CTD), and poly(A). The dual promoter that consisted of the CMV immediate early enhancer/promoter (pCMV) and the polyhedrin promoter (pPH) drove expression of the gene cassette.

Construction of BV-Dual-HA is shown in Figure 1; BV-Dual-HA harbored a gene cassette that consisted of the gp64 signal peptide, HA ectodomain gene, HA TM, CTD derived from gp64, and poly(A). The dual promoter that consisted of the CMV immediate early enhancer-promoter and the polyhedrin promoter drove expression of the gene cassette. Thus, a chimeric HA protein was designed to express both proteins on the viral envelope and in mammalian cells.

**Figure 2:**
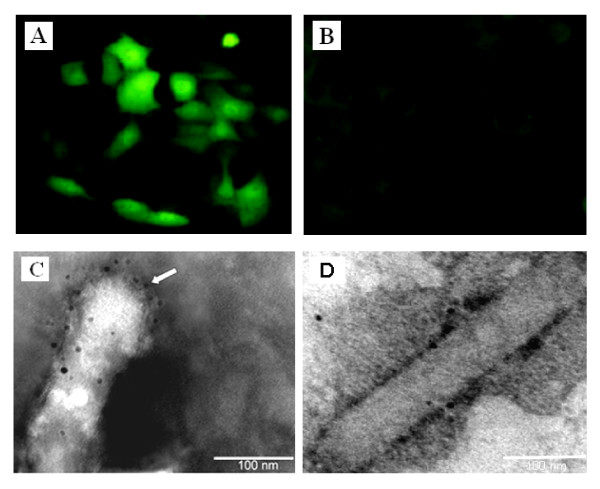
**Characterization of BV-Dual-HA**. PK-15 cells were transduced with BV-Dual-HA (A) or AcMNPV-WT (B) at an MOI of 10. At 48 h post-transduction, cells were fixed with absolute methanol, and processed for indirect immunofluorescent assay. Bound antibodies were detected by FITC-labeled anti-mouse IgG by fluorescence microscopy (green). Original magnification × 200. Electron micrograph of recombinant baculovirus displaying HA on the viral envelope. BV-Dual-HA (C) and AcMNPV-WT (D) were treated with anti-HA monoclonal antibodies, followed by labeling with anti-mouse IgG-gold conjugate. One end of the viral envelopes was strongly labeled with gold particles (arrows). Bars - 100 nm.

As shown in Figure 2A, the HA protein expressed with bright fluorescence could be detected by HA mAb in BV-Dual-HA transduced cells, but not in cells transduced with AcMNPV-Wt (Figure 2B), indicating that BV-Dual-HA can enter mammalian cells efficiently and express the HA protein.

To verify that the HA protein was displayed on the viral envelope, purified viral particles were analyzed by immunoelectron microscopy. As shown in Figure 2C, specific immunogold particles were evident on the surface of BV-Dual-HA, indicating the incorporation of chimeric HA and their display on the baculoviral envelope, whereas no gold particles were observed on the surface of AcMNPV-WT (Figure 2D). Moreover, incorporation of the chimeric HA did not alter virus morphology.

### Antibody responses in immunized mice

**Figure 3:**
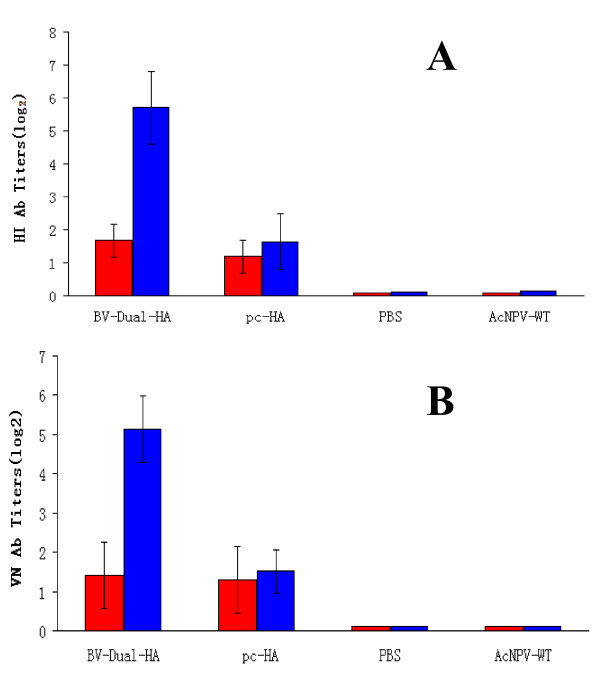
**Antibody responses in immunized mice with BV-Dual-HA**. Mice were immunized i.m. with BV-Dual-HA, pc-HA, AcMNPV-WT or PBS. An identical booster immunization was carried out 3 weeks later. Serum samples were collected on day 20 (red bars) and day 42 (blue bars) to determine the HI antibody titers (A), VN antibody titers (B). All data represent the mean ± S.D.

Immunization with BV-Dual-HA induced the highest levels of H9-specific HI antibodies and VN antibodies. At 20 days after primary immunization, all the mice immunized with BV-Dual-HA developed detectable H9-specific HI (Figure 3A) and VN antibodies (Figure 3B), while the titers were very low in mice immunized with pc-HA. Following a booster immunization, the mean titers of HI and VN antibodies increased greatly in mice immunized with BV-Dual-HA, and were significantly higher than those of the mice vaccinated with pc-HA (P < 0.01).

### Protection against challenge in vaccinated mice

**Figure 4:**
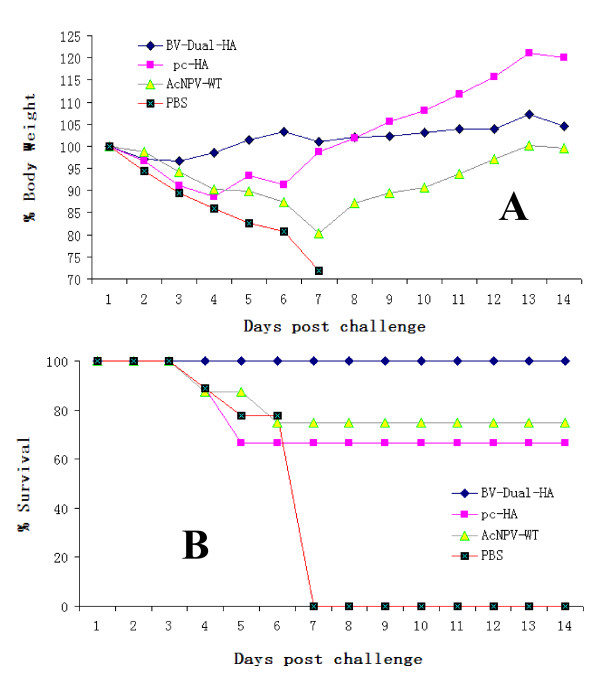
**Protective efficacy of BV-Dual-HA in mice**. Mean weight loss (A) and survival (B) were evaluated for 14 days after challenge. Mean weight loss is expressed as a percentage of original weight.

To evaluate the potency of the recombinant baculovirus BV-Dual-HA against lethal influenza virus challenge, the immunized mice were challenged with 50 MLD_50 _of H9N2 V strain on day 42. Starting from day 2 post-challenge, the mice immunized with pc-HA, AcMNPV-WT, or PBS displayed serious weight loss (Figure 4A) and signs of illness. In contrast, the BV-Dual-HA group only experienced slight weight loss shortly post-challenge. The survival rates in the BV-Dual-HA, pc-HA, and AcMNPV-WT groups at 14 days post-challenge were 100%, 66.7%, and 75%, respectively (Figure 4B).

To determine the virus titers in tissues, 3 mice in each group were sacrificed on day 6 post-challenge, and their lungs, brains, kidneys, and spleens were collected for analysis. As shown in Table 1, there were higher levels of virus titers in the lungs of the mice immunized with AcMNPV-WT or PBS, and virus titers were slightly lower in the mice immunized with pc-HA. Compared with those of the pc-HA group, no virus was detected in the lungs of mice immunized with BV-Dual-HA. The viral titers in the brains, kidneys, and spleens were also analyzed, but they were much lower than that in the lungs.

**Table 1:** Virus titration results of different tissue on day 6 post-challenge (log_10_EID_50_)^a^

Group	Virus titration results of different tissue on day 6 post-challenge (log_10_EID_50_)^b^			
	
	Brain	Lung	Kidney	Spleen
BV-Dual-HA	ND^c^	ND	ND	ND

pc-HA	ND	4.05 ± 0.54	ND	ND

AcMNPV-WT	ND	4.58 ± 0.38	1.25	ND

PBS	1 ± 0.78	5.17 ± 0.29	ND	ND

^a Each value represents the mean of 3 mice from each group.^

^b Results are expressed as the EID^_50 _^/0.2 mL and presented as mean ± S.D. for 3 mice.^

^c ND = no detection.^

## Discussion

In the present study, we developed a baculovirus dual expression system that possesses a gene cassette consisting of the chimeric HA gene under control of the CMV-polyhedrin dual promoter. We also investigated the efficacy of BV-Dual-HA as an avian influenza vaccine. Our results clearly show that immunization with BV-Dual-HA provided 100% protection, compared to the 66.7% and 75% protection observed with pc-HA and AcMNPV-WT immunization, respectively. After challenge, viral titers in the lungs, brains, kidneys, and spleens were determined on day 6 post-challenge. We found that all mice vaccinated with BV-Dual-HA had undetectable viral titers in these tissues, suggesting that antibodies induced by BV-Dual-HA conferred sterilizing immunity. Most mice vaccinated with pc-HA, AcMNPV-WT, or PBS had detectable lung virus titers by day 6 post-challenge. From these results, it is obvious that immunization with BV-Dual-HA can induce a robust antibody response and confer complete protection against lethal virus challenge in a mouse model, indicating that BV-Dual-HA is potential candidate vaccine that can prevent and control the pandemic spread of the H9N2 influenza virus.

BV-Dual-HA is capable of displaying HA protein on the surface of the viral envelope and expressing it upon transduction in mammalian cells, thus playing dual roles as a subunit/DNA vaccine, whereas pc-HA expressed HA and functioned like a DNA vaccine. Thus, the HA protein displayed on the envelope of BV-Dual-HA allowed it to engage antigen presenting cells (APCs) and activate the HA-specific immune reactions via the major histocompatibility complex II-mediated antigen presentation pathway, leading to more potent immune responses. Moreover, compared with the DNA vaccine, the baculovirus can directly transduce APCs [24,25] resident in the muscle tissues, especially more efficient antigen presentation to dendritic cells (DCs), which are the most important APCs [26]. Furthermore, Martyn et al. [27] reported that transduction by a recombinant baculovirus was more efficient than the transfection of conventional DNA plasmids driven by the CMV promoter in both cell lines and primary cells. The transduction of primary marmoset hepatocytes with recombinant baculovirus was 55 times more efficient than DNA transfection, highlighting a major advantage of recombinant baculovirus for the delivery of foreign genes to mammalian cells.

In this study, i.m. immunization of AcMNPV-WT alone provided 75% protection from the H9N2 influenza lethal challenge. This might be because the baculovirus envelope protein gp64 recognizes the TLR9 molecule and thus activates an innate immune response [28]. This is consistent with a previous study that determined that intranasal immunization with wild-type baculovirus alone also provides sufficient protection from the H1N1 influenza lethal challenge [29], though we didn't evaluate the cellular immune responses. And there are accumulated studies have demonstrated that the baculovirus itself has the ability to induce innate immune responses through a signaling pathway that is dependent on Toll-like receptor 9 (TLR9)/MyD88, which results in the production of various cytokines, including members of the IFN family [25,28,30]. Hervas-Stubbs reported that baculoviruses have strong adjuvant properties in mice, promoting potent humoral and CD8^+ ^T cell adaptive responses against co-administered antigens [30]. Besides, previously studies also have proved that baculovirus expressing PRRSV GP5 and M protein [31], PCV2 Cap protein [32], H5N1 virus HA protein [33], Pseudorabies virus glycoproteins[34] could induced a high level of IFN-g responses. The unique ability of the baculovirus to induce innate and adaptive immunity may have contributed to the protection from H9N2 influenza lethal challenge.

Accumulating evidence has shown that the H9N2 virus has undergone extensive reassortment, and novel genotypes have continued to emerge and evolve into several clades; this may increase the likelihood of avian-to-human interspecies transmission [10,14]. Thus, an ideal vaccine against the H9N2 avian influenza virus should overcome the antigenic variability of the virus. The baculovirus vector contains a large genome that enables insertion of large, foreign DNA fragments or the construction of multivalent vaccines. In subsequent studies, we plan to improve the protective range of this system using appropriate selection of HA genes derived from different clades of the H9N2 avian influenza virus for the composition of multivalent vaccines. In addition, we are also ready to co-express HA proteins and other immunogenic proteins of the H9N2 influenza virus, such as neuraminidase and M2, in order to enhance the immune response and protective efficiency.

## Conclusion

Our study provides an alternative method of applying the baculovirus dual expression system as an immunizing reagent against the influenza virus. Considering its safety and cost-effectiveness, a simple scale-up would be sufficient to produce a high-titer recombinant baculovirus, enabling BV-Dual-HA to be utilized as an alternative strategy to prevent and control the pandemic spread of the H9N2 influenza virus.

## Competing interests

The authors declare that they have no competing interests.

## Authors' contributions

WYL and HYF performed the experiments and wrote the manuscript, and should be considered as first authors. XLC, YY, XWC, TR, WBQ helped with the experiment. All authors read and approved the final manuscript. ML contributed to conceive the idea and initiate the project.

## References

[B1] Tang XY Tian GB Zhao CS Zhou JF Yu KZ Isolation and characterization of prevalent strains of avian influenza viruses in China Chin J Prev Vet Med 1998 20 1 5

[B2] Guan Y Shortridge KF Krauss S Webster RG Molecular characterization of H9N2 influenza viruses: were they the donors of the "internal" genes of H5N1 viruses in Hong Kong? Proc Natl Acad Sci USA 1999 96 9363 9367 10.1073/pnas.96.16.9363 10430948 PMC17788

[B3] Alexander DJ A review of avian influenza in different bird species Vet Microbiol 2000 74 3 13 10.1016/S0378-1135(00)00160-7 10799774

[B4] Munster VJ Fouchier RAM Avian influenza virus: Of virus and bird ecology Vaccine 2009 27 6340 6344 10.1016/j.vaccine.2009.02.082 19840670

[B5] Peiris M Yuen KY Leung CW Chan KH Ip PL Lai RW Orr WK Shortridge KF Human infection with influenza H9N2 Lancet 1999 354 916 917 10.1016/S0140-6736(99)03311-5 10489954

[B6] Guo Y Li J Cheng X Discovery of men infected by avian influenza A (H9N2) virus Chinese J Exp Clin Virol 1999 13 105 108 12569771

[B7] Peiris JSM Guan Y Markwell D Ghose P Webster RG Shortridge KF Cocirculation of avian H9N2 and contemporary "human" H3N2 influenza A viruses in pigs in southeastern China: potential for genetic reassortment? J Virol 2001 75 9679 9686 10.1128/JVI.75.20.9679-9686.2001 11559800 PMC114539

[B8] Saito T Lim W Suzuki T Suzuki Y Kida H Nishimura SI Tashiro M Characterisation of a human H9N2 influenza virus isolated in Hong Kong Vaccine 2001 20 125 133 10.1016/S0264-410X(01)00279-1 11567756

[B9] Choi YK Ozaki H Webby RJ Webster RG Peiris JS Poon L Butt C Leung YH Guan Y Continuing evolution of H9N2 influenza viruses in southeastern China J Virol 2004 78 8609 8614 10.1128/JVI.78.16.8609-8614.2004 15280470 PMC479067

[B10] Butt KM Smith GJ Chen H Zhang LJ Leung YH Xu KM Lim W Webster RG Yuen KY Peiris JS Guan Y Human infection with an avian H9N2 influenza A virus in Hong Kong in 2003 J Clin Microbiol 2005 43 5760 5767 10.1128/JCM.43.11.5760-5767.2005 16272514 PMC1287799

[B11] Li KS Xu KM Peiris JSM Poon LLM Yu KZ Yuen KY Shortridge KF Webster RG Guan Y Characterization of H9 subtype influenza viruses from the ducks of southern China: a candidate for the next influenza pandemic in humans? J Virol 2003 77 6988 6994 10.1128/JVI.77.12.6988-6994.2003 12768017 PMC156195

[B12] Xu KM Li KS Smith GJ Li JW Tai H Zhang JX Webster RG Peiris JS Chen H Guan Y Evolution and molecular epidemiology of H9N2 influenza A viruses from quail in southern China, 2000 to 2005 J Virol 2007 81 2635 2645 10.1128/JVI.02316-06 17192315 PMC1865985

[B13] Zhang P Tang Y Liu X Liu W Zhang X Liu H Peng D Gao S Wu Y Zhang L Lu S Liu X A novel genotype H9N2 influenza virus possessing human H5N1 internal genomes has been circulating in poultry in eastern China since 1998 J Virol 2009 83 8428 8438 10.1128/JVI.00659-09 19553328 PMC2738149

[B14] Sun Y Pu J Jiang Z Guan T Xia Y Xu Q Liu L Ma B Tian F Brown EG Liu J Genotypic evolution and antigenic drift of H9N2 influenza viruses in China from 1994 to 2008 Vet Microbiol 2010 146 215 225 10.1016/j.vetmic.2010.05.010 20685047

[B15] Hofmann C Sandig V Jennings G Rudolph M Schlag P Strauss M Efficient gene-transfer into human hepatocytes by baculovirus vectors Proc Natl Acad Sci USA 1995 92 10099 10103 10.1073/pnas.92.22.10099 7479733 PMC40743

[B16] Lu L Yu L Kwang J Baculovirus surface-displayed hemagglutinin of H5N1 influenza virus sustains its authentic cleavage, hemagglutination activity, and antigenicity Biochem Biophys Res Commun 2007 358 404 409 10.1016/j.bbrc.2007.04.148 17499217

[B17] Xu XG Liu HJ Baculovirus surface display of E2 envelope glycoprotein of classical swine fever virus and immunogenicity of the displayed proteins in a mouse model Vaccine 2008 26 5455 5460 10.1016/j.vaccine.2008.07.090 18708107

[B18] Yoshida S Kawasaki M Hariguchi N Hirota K Matsumoto M A baculovirus dual expression system-based malaria vaccine induces strong protection against Plasmodium berghei sporozoite challenge in mice Infect Immun 2009 7 1782 1789 10.1128/IAI.01226-08 PMC2681746 19223476

[B19] Yang DG Chung YC Lai YK Lai CW Liu HJ Hu YC Avian influenza virus hemagglutinin display on baculovirus envelope: cytoplasmic domain affects virus properties and vaccine potential Mol Ther 2007 15 989 996 10.1038/mt.sj.6300131 17375072

[B20] Tang XC Lu HR Ross TM Hemagglutinin displayed baculovirus protects against highly pathogenic influenza Vaccine 2010 28 6821 6831 10.1016/j.vaccine.2010.08.040 20727393

[B21] Feng Q Liu Y Qu X Deng H Ding M Lau TL Yu AC Chen J Baculovirus surface display of SARS coronavirus (SARS-CoV) spike protein and immunogenicity of the displayed protein in mice models DNA Cell Biol 2006 25 668 673 10.1089/dna.2006.25.668 17184168

[B22] O'Reilly D Miller L Luckow V Baculovirus expression vectors: a laboratory manual 1992 New York and WH Freeman and Co

[B23] Govorkova EA Webby RJ Humberd J Seiler JP Webster RG Immunization with reverse-genetics-produced H5N1 influenza vaccine protects ferrets against homologous and heterologous challenge J Infect Dis 2006 194 159 167 10.1086/505225 16779721

[B24] Abe T Hemmi H Miyamoto H Moriishi K Tamura S Takaku H Akira S Matsuura Y Involvement of the toll-like receptor 9 signaling pathway in the induction of innate immunity by baculovirus J Virol 2005 79 2847 2858 10.1128/JVI.79.5.2847-2858.2005 15709004 PMC548444

[B25] Abe T Kaname Y Wen X Tani H Moriishi K Uematsu S Takeuchi O Ishii KJ Kawai T Akira S Matsuura Y Baculovirus induces type I interferon production through toll-like receptor-dependent and independent pathways in a cell-type-specific manner J Virol 2009 83 7629 7640 10.1128/JVI.00679-09 19474102 PMC2708616

[B26] Strauss R Huser A Ni S Tuve S Kiviat N Sow PS Hofmann C Lieber A Baculovirus-based vaccination vectors allow for efficient induction of immune responses against *Plasmodium falciparum *circumsporozoite protein Mol Ther 2007 15 193 202 10.1038/sj.mt.6300008 17164791

[B27] Martyn JC Dong X Holmes-Brown S Pribul P Li S Drummer HE Gowans EJ Transient and stable expression of the HCV envelope glycoproteins in cell lines and primary hepatocytes transduced with a recombinant baculovirus Arch Viro 2007 152 329 343 10.1007/s00705-006-0845-5 17019531

[B28] Abe T Matsuura Y Host innate immune responses induced by baculovirus in mammals Curr Gene Ther 2010 10 226 231 10.2174/156652310791321279 20394574

[B29] Abe T Takahashi H Hamazaki H Miyano-Kurosaki N Matsuura Y Takaku H Baculovirus induces an innate immune response and confers protection from lethal influenza virus infection in mice J Immunol 2003 171 1133 1139 12874198 10.4049/jimmunol.171.3.1133

[B30] Hervas-Stubbs S Rueda P Lopez L Leclerc C Insect baculoviruses strongly potentiate adaptive immune responses by inducing type I IFN J Immunol 2007 178 2361 2369 17277142 10.4049/jimmunol.178.4.2361

[B31] Wang SP Fang LR Fan HY Jiang YB Pan YF Luo Q Chen HC Xiao SB Construction and immunogenicity of pseudotype baculovirus expressing GP5 and M protein of porcine reproductive and respiratory syndrome virus Vaccine 2007 25 8220 8227 10.1016/j.vaccine.2007.09.069 17980465

[B32] Fan HY Pan YF Fang LR Wang D Wang SP Jiang YB Chen HC Xiao SB Construction and immunogenicity of recombinant pseudotype baculovirus expressing the capsid protein of porcine circovirus type 2 in mice J Virol Methods 2008 150 21 26 10.1016/j.jviromet.2008.02.011 18394722

[B33] Wu Q Fang L Wu X Li B Luo R Yu Z Jin M Chen H Xiao S A pseudotype baculovirus-mediated vaccine confers protective immunity against lethal challenge with H5N1 avian influenza virus in mice and chickens Mol Immunol 2009 13 2210 2217 10.1016/j.molimm.2009.04.017 19446339

[B34] Grabowska AK Lipińska AD Rohde J Szewczyk B Bienkowska-Szewczyk K Rziha HJ New baculovirus recombinants expressing Pseudorabies virus(PRV) glycoproteins protect mice against lethal challenge infection Vaccine 2009 27 3584 3591 10.1016/j.vaccine.2009.03.067 19464538

